# Recruitment and retention strategies to promote research engagement among caregivers and their children: A scoping review

**DOI:** 10.1017/cts.2024.624

**Published:** 2024-11-08

**Authors:** Tammy E. Corr, Alma Jusufagic, James Basting, Catherine Caldwell, Steven King, Aleksandra E. Zgierska

**Affiliations:** 1 Department of Pediatrics, Division of Neonatal-Perinatal Medicine, Penn State College of Medicine, Penn State Milton S. Hershey Medical Center, Hershey, PA, USA; 2 Department of Obstetrics and Gynecology, MedStar Georgetown University Hospital and Washington Hospital Center, WA, District of Columbia, USA; 3 Department of internal Medicine, University of Kentucky, Lexington, KY, USA; 4 Department of Neurosurgery, University of Kentucky, Lexington, KY, USA; 5 Department of Internal Medicine, University of Michigan Health, Ann Arbor, MI, USA; 6 Departments of Family and Community Medicine, Public Health Sciences, and Anesthesiology and Perioperative Medicine, Penn State College of Medicine, Penn State Milton S. Hershey Medical Center, Hershey, PA, USA

**Keywords:** Recruitment, retention, mothers, caregivers, children

## Abstract

Long-term health and developmental impact after *in utero* opioid and other substance exposures is unclear. There is an urgent need for well-designed, prospective, long-term observational studies. The HEALthy Brain and Child Development Study aims to address this need. It will require optimizing recruitment and retention of caregivers and young children in long-term research. Therefore, a scoping review of original research articles, indexed in the PubMed database and published in English between January 1, 2010, and November 23, 2023, was conducted on recruitment and retention strategies of caregiver–child (≤6 years old) dyads in observational, cohort studies. Among 2,902 titles/abstracts reviewed, 37 articles were found eligible. Of those, 29 (78%) addressed recruitment, and 18 (49%) addressed retention. Thirty-four (92%) articles focused on strategies for facilitating recruitment and/or retention, while 18 (49%) described potentially harmful approaches. Recruitment and retention facilitators included face-to-face and regular contact, establishing a relationship with study personnel, use of technology and social platforms, minimizing inconveniences, and promoting incentives. This review demonstrates that numerous factors can affect engagement of caregivers and their children in long-term cohort studies. Better understanding of these factors can inform researchers about optimal approaches to recruitment and retention of caregiver–child dyads in longitudinal research.

## Introduction

For nearly two decades, rates of both maternal opioid use-related diagnoses and neonatal opioid withdrawal syndrome (NOWS, formerly known as neonatal abstinence syndrome) have continued to rise [1–3]. Long-term health and developmental outcomes of children exposed to *in utero* opioids are unclear, but several studies suggest that children with prenatal opioid exposure remain at increased risk for future adverse health [4–8], behavioral [9–12], speech-language [10,12–14], and academic outcomes [14,15]. There is an urgent need for well-designed, prospective, long-term cohort studies to evaluate these findings and the impact of early opioid and other substance exposure on later childhood outcomes.

Understanding the health and neurodevelopmental consequences of *in utero* substance exposure on children will allow for the development of targeted interventions to improve the long-term well-being and success of these children. Previous work on NOWS outcomes has been criticized for not accounting for the effects of socioenvironmental factors on child health and development, prompting content experts, the National Institutes of Health (NIH), and the Substance Abuse and Mental Health Services Administration (SAMHSA) to call for research evaluating prenatal substance exposure-related outcomes while accounting for highly influential variables [16–19]. As such, the NIH Helping to End Addiction Long-term® (HEAL) HEALthy Brain and Child Development (HBCD) study aims to apply a multidimensional assessment of perinatal factors that may impact a child’s health and development, including, but not limited to, prenatal substance exposure. It will include collection of medical and family histories; behavioral and developmental evaluations; structural and functional brain assessments (magnetic resonance imaging, and electroencephalography); biospecimen collection; and social and home environment evaluation in order to identify risk and resilience factors that may mitigate adverse outcomes [20]. The HBCD birth cohort study plans to accomplish this aim through the recruitment of a large sample of pregnant and early postpartum persons across the USA and follow-up of the child–caregiver dyads through early childhood.

Recruitment and retention of pregnant individuals and their children into research can be challenging [21], and prenatal substance exposure further increases the complexity of this research engagement. Individuals with substance use disorders (SUD) often experience socioeconomic difficulties, including unemployment, housing insecurity, transportation barriers, societal stigma, and concurrent mental health issues [22–24], all of which can make involvement in longitudinal research difficult [25]. Additionally, fear of child loss, Child Protective Service involvement, and legal repercussions are specific concerns faced by pregnant persons with SUD [22–24,26], and make this population particularly vulnerable when approaching them for research involvement. Understanding the additional challenges that may arise in recruiting caregivers with SUD, this study aimed to offset these challenges by summarizing what is already known about factors promoting research engagement among caregivers and their children in longitudinal studies in preparation for the HBCD study [20].

## Methods

The authors followed the Preferred Reporting Items for Systematic reviews and Meta-Analyses Extension for Scoping Reviews guidelines [27]. The PubMed search engine was used to perform a literature review restricted to articles published from January 1, 2010, through November 23, 2023, in order to focus on modern approaches to participant engagement and because metanalyses and systematic reviews of recruitment and retention published before 2010 are extant in the literature [28,29]. Relevant search terms related to caregivers and/or children were combined first with the term “recruitment” and then “retention” using the following format: (search term) AND (recruitment) or (retention), (Supplementary Table 1).

**Figure 1.**
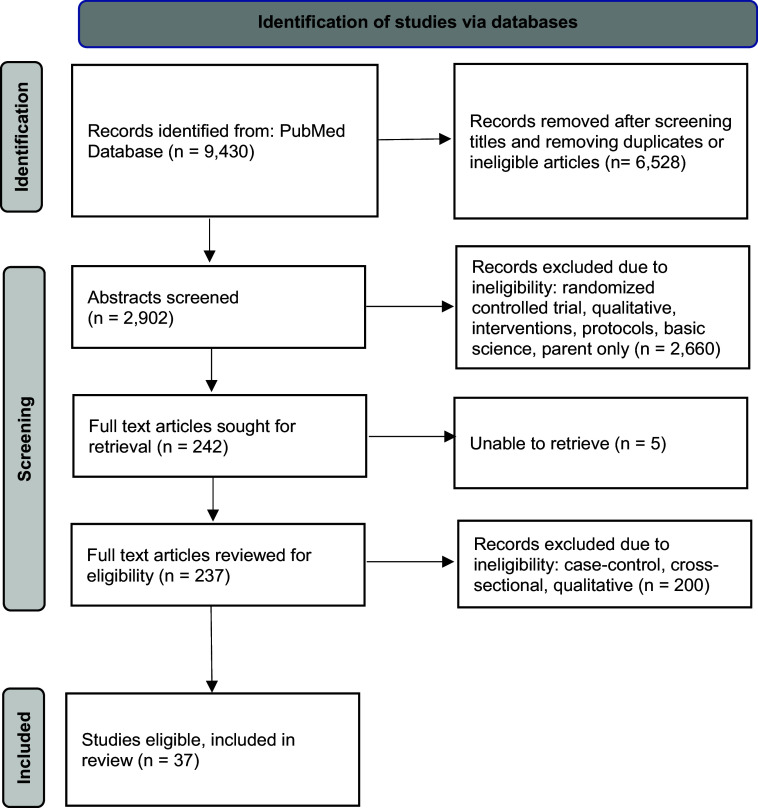
Preferred reporting items for systematic reviews and meta-analyses (PRISMA) flow diagram.

Eligible articles were published as full text in the English language and described recruitment and/or retention-related findings in longitudinal, observational cohort studies with at least two distinct data collection points involving caregiver–child dyads with children 6 years of age and younger. Titles and abstracts procured using the prespecified search terms were reviewed by five authors (TEC, AJ, JB, CC, and SK) to remove duplicates and ineligible articles. Full-text articles of potentially eligible manuscripts were then reviewed independently for inclusion (Fig. 1). Articles of unclear eligibility were discussed among the authors, and all discrepancies (*n* = 12) were resolved via consensus.

Data extraction from the included articles was completed by the aforementioned authors and included systematic extraction of information on the study design, goals, participant data collected, population, follow-up duration, percent enrolled and percent retained, and outcomes related to recruitment and retention facilitators and barriers. These data are presented in Table 1 for each included article and summarized across all articles in the text. Given the methodological heterogeneity of the included studies, a formal data pooling and metanalysis were not possible.

**Table 1.** Article summaries

Author	Study Focus	Participant Data Collected	Population	Length of Follow-Up	Recruitment Rate	Retention Rate	Comments
Grech et al. (2023)	Identify factors in pregnancy and early life that impact long-term health; assess acceptance of longitudinal birth cohort study (BABY1000 study)	Biospecimens (saliva, stool, blood, buccal); anthropometrics; sociodemographics; medical/psych history; child development	225 pregnant or preconception women and their newborns	10-12, 20, 28, and 36 weeks gestation; birth, 6 weeks; data collection thru 2 years planned	N/A	95% at 12 weeks 71% at 20 weeks 76% at 28 weeks 74% at 36 weeks 80% at birth 45% at 6 weeks	Data collection using primarily non-invasive procedures, coordinated with routine healthcare, maximized data quality and participant retention
Leonard et al. (2023)	Prospectively study of autoimmune conditions & the role of the environment in the development of celiac disease in at-risk children (CDGEMM^a^ study)	Stool & blood samples; growth measurements, questionnaires re: the participant, family, and environment	554 pregnant women & infants up to 6 months with first-degree relative with celiac disease	Birth, 7-10 days, every 3 months for the first 3 years, every 6 months until 5/10 years	Not reported	82% from US & 70% from Italy; study ongoing	Online networking was a strong source of recruitment; communication & feeling involved crucial for retention (e.g. Facebook group, cards, child completion certificate)
Li et al. (2023)	Identify factors associated with research participation & withdrawal in a children’s primary care cohort study (TARGet Kids!^b^)	Questionnaires and physical measures	10,412 children less than 5 years and their parents/caregivers	2x/year thru 2 years; annually at well child visit thru 17 years old	N/A	68% attended at least 1 follow-up visit	Most frequent reason for study withdrawal was too time consuming; retention strategies included engaging parent, avoiding question redundancy
Ozonoff et al. (2023)	Examine child/family factors associated with retention in a longitudinal study of siblings of children with autism spectrum disorder (ASD) or typical development	Demographics, parent interviews, developmental assessments, autism diagnostic observation schedule (ADOS-2^c^)	304 siblings of children with ASD & their parents; 163 siblings of typical development & their parents	First year to 36 months for up to 7 visits (6, 9, 12, 15, 18, 24, & 36 months)	83%	85% at 36 months	Greater travel distance associated with lower retention; more contacts with families on enrollment & scheduling associated with greater retention
McGorm et al. (2022)	Compare characteristics & retention of Facebook vs conventional recruits for the Environmental Determinants of Islet Autoimmunity (ENDIA) birth cohort study	Biospecimens (blood, urine, stool, swabs); lifestyle & dietary questionnaires	1,473 pregnant or delivered women with an infant<6 months with a first degree relative with type 1 diabetes	Every 3 months from pregnancy to 2 years; then every 6 months to adolescence	1,511 recruited resulting in 1,473 lives births; recruitment rate not reported	88% of conventional & 95% of Facebook recruits at 3.3 years	Facebook was the third largest referral source (20%); Facebook recruits more often enrolled postnatally & were 3x less likely to withdraw vs conventional recruits
Sarfi et al. (2022)	Describe parenting stress, child behavioral problems, & postnatal mental health of mothers in opioid maintenance therapy (OMT) vs comparison	Parenting stress questionnaires, Edinburgh postnatal depression scales, child development and behavior checklists	36 pregnant OMT mothers and their infants; 36 age-matched pregnant women and their infants	3 & 6 months, 1 & 2.5 years, 4.5 years, 8 years	76% of OMT group; comparison group not reported	72% OMT group & 67% in comparison group at 8 years	Maternal responses were not shared with treatment providers or child welfare services, resulting in an excellent working alliance & high retention rate
Sobotka et al. (2022)	Provide details of follow-up methodology in a PICU survivor cohort	Surveys/validated assessment tools on resource utilization, family impact, and child function and neurodevelopment	152 children aged 0 to 17 years admitted to the PICU and their parents/guardians	1, 3, 6 months & 1 year after discharge & then yearly thru 3 years	70%	89, 78, 83% & 80, 55, 43% at 1, 3, & 6 months & 1, 2, & 3 years	Cohort retention necessitated persistent contact attempts, incentives, flexibility, & attention to study burden; despite this effort, retention decreased beyond year 1
Song et al. (2020)	Describe methodological challenges in conducting grandparent–grandchild dyad research, particularly when grandparents are caregivers to the child participants	Dietary behaviors, physical activity, sedentary time, body mass index, waist-to-height ratio	9 grandparents aged≥50 years and their grandchildren aged 6–12 years	8 weeks	7%	100%	Low recruitment rate (9/124) due to ineligibility rather than lack of interest; offering different options for questionnaires & communication helped engage grandparents
van Gelder et al. (2020)	Evaluate recruitment & data collection methods in a pregnancy/early life exposure study on short & long term health of mother & child (PRIDE^d^ study)	Serial questionnaires & saliva sample for all; biospecimens (blood: mother, feces: mother & infant) for subgroup	8,360 pregnant women and their newborns	17 and 34 weeks of pregnancy; 2 & 6 months of age; biannually to 21 years old	Not calculated due to diversity of recruitment methods & lack of valid denominator	70% at 6 months post estimated date of delivery	Increased diversity with addition of Facebook Ads for recruitment (younger, lower education, & primiparous); using web-based surveys also increased diversity
Wong et al. (2020)	Describe a study approach, using efficient processes for biological and clinical data collection, to feasibly establish a preterm cohort with genetic specimens	Genotyping, neonatal clinical data, and longitudinal health and educational data	779 very preterm infants and their parents	2 years for initial health data; not specified for longitudinal data	42% approached; 19% of eligible population	90% agreed to follow-up; study ongoing	Utilized existing national health database for data collection, opportunistic blood sampling, & prolonged recruitment period after birth to minimize family burden
Barry et al. (2019)	Provide an overview of a cohort management platform (CMP) design used in a large, longitudinal birth cohort study (NEST^e^)	Survey questions and data collection on exposures and health outcomes; biological sample	2,595 mothers and their children in original study; 302 approached about CMP	Goal = 18 years	99% (298) agreed to participate in CMP	N/A; ongoing	Used flexible, cloud-based communication system via interactive voice response & SMS to maximize retention and assist in data collection
Brand et al. (2019)	Explore the feasibility of recruiting children from playdates in a research laboratory	Not specified; focus on converting playdates to research participants in developmental studies	33 parents and their babies and/or toddlers	Not disclosed; participants recruited to several, different studies	33% enrolled; 88% agreed to be contacted (awaiting age eligibility); 81% of these returned	N/A	Playdate method doubled participant recruitment rates as compared to calling
Gordon et al. (2019)	Describe outcomes of a chart review recruitment method on enrollment, scheduling, and attrition	Microbiome data	25 preterm infants and their parents	Toddler & preschool age	53%	92%	Phone calls & Facebook result in better participant response than postcards; Facebook & online modalities are less intrusive & allow participant control
Stephenson et al. (2019)	Determine if Facebook is a feasible method to identify and reengage participants of a longitudinal cohort who were lost to follow-up	Maternal and cord blood sampling; questionnaires at every visit	237 mother-child dyads who were lost to follow-up of 2,827 dyads enrolled	22-24 & 32-36 weeks of pregnancy; 4 months and 1, 2, 3, & 5 years	N/A	92% at 5-year evaluation	Facebook identified 48% of participants who were lost to follow-up; contact was made in 37%; reengagement was successful in 8%
Stroustrup et al. (2019)	Evaluate impact of neonatal intensive care unit exposure on early life development	Biospecimen collection, observational + survey data, comprehensive multisystem outcomes	Goal is 400 moderately preterm infants and their mothers (ongoing)	6 visits in first 2 years; planned follow-up at ages 3-12 years	N/A	77% to date	Detailed surveys & assessments were conducted while infant was hospitalized so early absent data were low
van Gelder et al. (2019)	Evaluate online vs provider recruitment in a study identifying exposures in pregnancy and early life that affect short- & long-term health of mother & child (PRIDE^d^ study)	Serial questionnaires and saliva sample for all; blood from mother, feces from mother/infant for subgroup	392 pregnant women and their newborns	17 & 34 weeks of pregnancy; 2 & 6 months of age; biannually to 21 years old	Not reported	66% in Facebook Ads group; 75% in prenatal care provider group	Recruitment via Facebook Ads added diversity (younger, higher BMI’s, and low/intermediate education) and complemented traditional recruitment methods
Bartlett et al. (2018)	Evaluate parental reasons for participating in/declining a longitudinal natural history study of infants with spinal muscular atrophy (SMA); prep for SMA clinical trial	Infant motor function scales, physiologic and molecular biomarkers, parental survey	26 SMA infants<6 months and their families; 27 healthy control infants and their families	6, 9, 12, 18, and 24 months of age. Parental survey at 9-month visit	98%; 72% of enrolled participated in recruitment survey	29% SMA (46% had died at time of survey); 85% of controls	The most common reason for a family to decide to enroll their infant (58%) and to remain in the study (74%) was their understanding of the importance of the study
Ishikuro et al. (2018)	Establish strategies to recruit extended family members, focusing on grandparents, for the Tohoku Medical Megabank Birth and Three-Generation Cohort Study	For all generations: questionnaires on lifestyle, medical history, SES^f^, mental health; health records; health assessments	Pregnant women & their children, partners, parents; this subanalysis: 8,054 grandparents	6, 12, 24, 36, 42, 48, 60 months and then yearly for children; adults “similar” methods	69%	N/A	Invitation letters are helpful in recruiting grandparents; grandparents more frequently participated in study if father also participating
Bergmann et al (2017)	Investigate recruitment strategies in a longitudinal family obesity study for cost effectiveness and enrollment	Unclear; weight and growth measurements	178 families with at least one obese parent and their children 6-47 months	Not specified	30%	N/A	Low recruitment due to ineligibility, not disinterest; community-focused recruitment most successful – 37% of sample
Parent et al. (2017)	Examine inclusion of fathers in child psychopathology research and examine online crowdsourcing as a method to recruit fathers	Surveys on demographics, child behavioral problems, co-parenting conflict, and parenting practices	564 parents with a child 3-17 years of age	2 weeks, 4 months, 8 months, and 12 months	Not reported	Fathers: 75, 62, 55%, & 58% at 2 weeks and 4, 8, and 12 months	Fathers more likely to drop out over 12-month follow-up but differences between mothers and fathers were nonsignificant if fathers were retained at 2 weeks
Park et al. (2017)	Compare household, provider, direct-outreach and provider-based sampling (PBS) in recruitment success, cost, efficiency and study goals; National Children’s Study	Survey/interview data, biologic and environmental specimens	6,897 women of child-bearing age and their newborns (goal 100,000; study closed)	Goal = 21 years	71% of eligible women consented; of these women, 87% enrolled their newborns	N/A; study closed	PBS had an observed: expected newborn enrollment ratio of 0.99 vs other strategies (0.35-0.48); study ultimately closed due to unworkable models and study infeasibility
Thibadeau et al. (2017)	Assess the development, health, and condition progression of children born with spina bifida and evaluate the methodology used for data collection	Medical history; report of family functioning, child behaviors, self-care, mobility, and well-being; and child neuro-psychological testing	101 children with spina bifida aged 3-6 years and their parents	Not reported	66%	N/A, study ongoing	To reduce burden of in-person assessment, a partial protocol that involved mailed surveys only was offered; 20% of participants opted for this approach
Ballieux et al. (2016)	Explore the feasibility of recruiting children from low SES^f^ into studies of neurocognitive function through child-care centers	Eye-tracking assessments	174 infants aged 6-8 months and their parents	18 months	∼ 50% (range 33-65% across child care centers)	“More than half;” number not specified	Completing studies in child care centers may facilitate recruitment and assessment of large samples of infants from diverse SES^f^ & ethnicities
Daly et al. (2016)	Determine which recruitment methods most effectively resulted in enrollment of parent/infant pairs	Dental screening examinations and parental questionnaires	348 mothers and their 12-month-old infants	30 and 48 months of age	68%	N/A	Face-to-face strategies had the highest recruitment rates (25%); mass email had the lowest rates (0.09%) but produced 6% of enrollees with minimal effort
Johnson et al. (2016)	Identify predictors of later study withdrawal among participants in The Environmental Determinants of Diabetes in the Young (TEDDY) study for≥1 year	Blood draws; interviews and questionnaires on diet, health, stressors, and environmental exposures	8,627 infants age<4.5 months with HLA^g^-conferred Type 1 diabetes genetic risk and their parents	Every 3 months to age 4 years and then every 6 months up to 15 years	Not reported	Not reported	Modifiable psychosocial and behavioral factors, such as maternal lifestyle behaviors, mother’s risk perception, and study engagement, predicted late study attrition
Woolfenden et al. (2016)	Describe the establishment of a birth cohort, recruitment processes, representativeness and follow-up	Questionnaire results, medical record data	2,025 newborn infants and their parents	6, 12, and 18 months of age	50%	39, 32%, & 28% at 6, 12, and 18 months, respectively	Greater enrollment with a researcher, which allowed dual discussion and consent; nurse recruitment was lower, presumably because of their inability to obtain consent during study discussion
Robbins et al. (2015)	Describe outcomes, successes, and challenges of recruiting women through prenatal care providers (prework for the National Children’s Study)	Longitudinal data: survey/interview data, biologic and environmental specimens	151 women of child-bearing age and their newborns (goal 100,000; study closed)	Pregnancy, birth, and 3, 6, 9, 12, 18, and 24 months postpartum; goal = 21 years	68%	95% over first 2 years; study closed before completion	Enrolling pregnant women from prenatal care providers can be efficient; study ultimately closed due to unworkable models and study infeasibility
Maghera et al. (2014)	Compare recruitment rates and demographic differences between recruitment methods for simultaneous recruitment in 1 of 4 pregnancy cohort studies	Varied per study: nutrition/development, genetic/environmental influences and develop-ment, chemicals, stress/development	982 pregnant women and their children	Varied based on study	31%	N/A	Recruitment methods result in different recruitment rates and participant demographics; a variety of methods are required to recruit a generalizable sample
Brannon et al. (2013)	Describe strategies to minimize recruitment and retention barriers in African-American families with low SES^f^ in a child weight study	Demographics, brief interview, anthro-pometric measures, dual X-ray absorptionmetry (DEXA) scan, food recall summary	76 African-American families with children aged 36–59 months	1 and 2 years after initial encounter	66%	66% at 1 year; 46% at 2 years	Community involvement, budgeting for supports for low-SES^f^ families and ethnic minorities, planning, and building rapport were credited for R&R^h^ success
Chudleigh et al. (2013)	Assess the feasibility of recruiting and retaining infants with cystic fibrosis (CF) and healthy controls to a longitudinal, observational study	Pulmonary function tests, chest computed tomography, and bronchoscopy and bronchioalveolar lavage data	92 families and their children with CF; 77 healthy controls	3 and 12 months of age	86% for CF; 29% for healthy controls	CF: 92% & 91% at 3 & 12 months, respectively; controls: 73% & 49% at 3 & 12 months	“Helping my/other child(ren)” was the most common reason parents chose to have their child participate in research
Fahrenwald et al. (2013)	Describe community outreach and engagement in preparation for recruitment of women at a rural location of the National Children’s Study	Longitudinal data: survey/interview data, biologic and environmental specimens	5,800 women of child-bearing age (18-49 years) identified as potential candidates	Goal = 21 years	Screening interviews were conducted with 89% of age-eligible women	N/A; study closed	1-2 years of effort prior to active recruitment suggested when working with rural health facilities; study closed due to unworkable models and study infeasibility
Manca et al. (2013)	Describe recruitment strategies and discuss reasons for different success rates between 2 cities in the Alberta Pregnancy Outcomes and Nutrition (APrON) study	Questionnaires; biologic specimens (maternal blood, urine, DNA^i^, breastmilk); child DNA and neuro-cognitive evaluation; anthropometrics	1,200 pregnant women<27 weeks and their children	Each trimester, twice after delivery, five questionnaires through 3 years of age	Not reported	N/A	Recruitment in high-volume offices was most successful and economical when clinic staff discussed study with patients and sent contact info to research team rather than having a recruiter in clinic
Riesch et al. (2013)	Describe and evaluate outreach and engagement strategies designed to build awareness for the National Children’s Study	Longitudinal data: survey/interview data, biologic and environmental specimens	N/A – study prework to build awareness	Goal = 21 years	N/A – focus was bringing aware-ness of the study to communities prior to recruitment	N/A	Mailings were most common source of study awareness (64%); awareness highest in areas with higher median per capita income/median home value and more married men
Menon et al. (2012)	Describe associations among consent rates in pediatric critical care research and factors related to patients, legal guardians, consent processes, and study design	Demographic data, reason for consent refusal, perception of parental anxiety	217 pediatric intensive care unit (PICU) patients and their legal guardians	Varied; included analysis of consent in 45 Canadian PICU studies	80%	N/A	Higher recruitment rates when research assistant was introduced by a clinician before approaching family; association between parental anxiety and lower consent rate
Greeley et al. (2011)	Describe development of monogenic diabetes registry for longitudinal research recruitment of persons with maturity-onset diabetes of young or neonatal diabetes	Surveys on diagnosis, treatment, genetics; medical records on clinic visits, labs, quality of life, and neurodevelopment	727 children with neonatal diabetes or maturity-onset diabetes of the young and their parents	Annually; no end-point specified	Not reported; ongoing enrollment	N/A	Early, the majority found the study independently; over time, increased physician referral as registry became known as major center for studying monogenic diabetes
Phillips et al. (2011)	Examine participation barriers for Latinas and devise possible solutions to overcome these barriers in a longitudinal, iron-deficiency study	Medical record data, umbilical cord blood, infant blood sampling	255 mothers and their full-term infants	Follow-up outpatient infant blood sample; timing not specified	60% overall; only 8% of the enrollees were Latina while 16.3% was anticipated	N/A	Improved Latina recruitment to 20% of enrollees thru increased identification of Latinas, increased recruiter hours, earlier family involvement in consent, and increased use of interpreters
Zook et al. (2010)	Describe retention strategies and challenges in the Urban Environment and Childhood Asthma (URECA) study; examine how maternal traits relate to retention	Cord blood; yearly physical exam, growth measurements, blood and nasal samples, dust and air samples; interviews	606 pregnant women with a self or paternal history of asthma or allergic rhinitis and their infants	Age 3 months and then yearly in home/clinic until 7 years; phone calls every 3 months	N/A	89% at 2 years	The most common reason for loss of follow-up was out-of-date contact information; obtaining several alternative contacts was helpful

a CDGEMM = Celiac Disease Genomic Environmental Microbiome and Metabolomic Study; ^b^TARGet Kids! = The Applied Research Group for Kids Study; ^c^ADOS = Autism Diagnostic Observation Schedule-2; ^d^PRIDE = PRegnancy and Infant DEvelopment Study; ^e^NEST = The Newborn Epigenetic STudy; ^f^SES = socioeconomic status; ^g^HLA = human leukocyte antigen; ^h^R&R = recruitment and retention; ^i^DNA = deoxyribonucleic acid.

## Results

### Article selection

The PubMed database search identified 9,430 articles. After review of titles and removal of duplicates, 2,902 abstracts remained and were reviewed. After application of the exclusion criteria, 237 articles remained for full-text review. Of these manuscripts, 37 met the eligibility criteria and were included in this review (Figure 1).

### Characteristics of included studies

Twenty-nine (78%) manuscripts addressed recruitment while 18 (49%) addressed strategies for retention. Thirty-four (92%) articles discussed approaches that support recruitment and/or retention and 18 (49%) addressed factors that hinder recruitment/retention.

The 37 articles included described 34 separate cohort studies from 8 different countries (USA, UK, Australia, Italy, Netherlands, Canada, Germany, and Japan). Results from some studies were featured in several articles (e.g., National Children’s Study [NCS] [30–33] and PRegnancy and Infant DEvelopment Study [PRIDE] [34,35]). Methodologies of the studies described within the 37 included articles were heterogeneous in terms of the study goals, length of follow-up, and data collected, and the study sample sizes ranged from 9 to 10,412 participants.

Sixteen articles focused on newborns and their parents, and 12 of those studies approached adults who were pregnant or in the preconception period. Most articles (*n* = 18) aimed to recruit mothers and their children, while 10 manuscripts were inclusive of either parent and their offspring, and 4 more broadly targeted children and their families. Fewer articles specifically addressed fathers (*n* = 1) or grandparents (*n* = 1), and only three articles more broadly focused on legal guardians/caregivers. While all articles included children ≤ 6 years per the review’s eligibility criteria, the age of the children in each study varied, spanning from *in utero* to 17 years. Three articles addressed underrepresented minorities, and two focused on families from lower socioeconomic backgrounds.

The duration of follow-up ranged from toddlerhood to a goal of 21 years, though one of these prolonged-monitoring studies closed prematurely due to lack of feasibility (National Children’s Study) [30–33] and the other is still ongoing, approaching completion of its first decade (PRegnancy and Infant DEvelopment [PRIDE] Study) [34,35]. The number of data collection time points varied from as few as two [36,37] to 46 different family interactions in the aforementioned PRIDE study [34,35]. Types of data collected included questionnaires on demographics, diet, health, education, stressors/conflict, family functioning, environmental exposures, and behavior; biospecimen samples of blood, cord blood, saliva, feces, breastmilk, and cheek swabs; environmental specimens of air, dust, and water; child growth measurements and physical exams; and child evaluations such as assessment of imaging, microbiome, genotyping, motor function, eye-tracking, neuropsychological testing, and developmental performance.

### Recruitment and retention considerations

#### Setting of recruitment

Several factors appeared to influence research recruitment and retention for caregivers and their children. In-person, face-to-face recruitment was found to be very effective [38–41] and may even increase retention and benefit the study long term [35,42], but it was both labor-intensive and costly. These factors may lead investigators to consider other less time-consuming approaches [31,39,43], such as mass emailing [39] or paid media advertisements like commercials, brochures, or radio advertisements, despite relatively lower enrollment rates with these methods [38,44]. Other wide-reaching, web-based tools, such as social media platforms [38,45–48], online advertisements and programs [35,45], and web-based questionnaires, [34] were also found to be valuable approaches for recruiting caregivers and their children into research studies and supported family convenience for data collection. Postal mailing and telephone calls were generally found to result in lower participant recruitment and were toilsome [46].

Mailing letters did have its place; however, in raising awareness of a research investigation in a community [32], and community-based recruitment, including posting flyers in public places and in-person recruitment at shopping centers, festivals, parades, and other public events, were effective strategies [43], particularly in rural neighborhoods [30]. Finally, creating disease-specific registries was useful in increasing knowledge of research investigations and in helping caregivers find available, relevant studies [49].

#### Personnel recruiting

Equally important to the setting of participant recruitment was the personnel involved in the recruiting. Medical provider-based sampling achieved high recruitment success [31]. However, this approach can be burdensome for the clinician and reduce referrals, particularly when the task was perceived as interfering with competing workloads or the clinician–patient relationship [44,50]. There are strategies that could be employed by the study team to minimize this burden. For example, establishing a relationship and workflow with the referring clinicians [33,51], and seeking referrals without placing the burden of consent on the provider [44,52] were effective in keeping the participating clinician engaged and facilitated recruitment. Other strategies to increase success of enrollment in healthcare settings included designating “a champion” among healthcare personnel to communicate with prospective participants [53] and be solely dedicated to recruitment [51]. Providing reading material in the waiting room followed by a brief discussion and referral by the clinician were likewise effective, capitalizing on patient–clinician relationship and provider involvement [44].

Although recruitment directly by the researcher can be efficient, recruitment success can be increased when the research is first introduced to the caregiver by a member of the child/family’s healthcare team [38,41,54]. However, this approach may not be universally effective as evidenced by one study that found that using clinical nurses to explain the study followed by referral of interested patients to the research team yielded such low response rates that the research staff turned to approaching caregivers directly [54]. Researchers recruiting in the healthcare setting may be especially successful enrolling pregnant persons and their children as the recurrent obstetrical visits afford multiple opportunities for contact [40].

Other approaches to study enrollment include partnering with nontraditional recruitment personnel. For example, utilizing staff at childcare centers was found to be a useful approach to recruiting children and their guardians into research studies [36]. For caregivers with children who do not participate in formal childcare, playdates at the research lab, where caregivers could socialize with babysitters available to care for the children, were a unique and successful strategy for recruiting young children into investigations [55].

#### Relationship with study staff

Similar to recruitment, retention of research participants often depended on the caregiver establishing a relationship with the study team [53]. This connection can be fostered by assigning a dedicated study coordinator [51] and building a team of well-trained research staff [38]. Additionally, experience of the study team appeared to positively influence participant retention over time, presumably through a more skilled approach and better rapport with study participants [56].

Sustaining relationships over time was facilitated by repeated contact with participants between study visits and was shown to improve retention and follow-up [57], while prolonged time between visits and lost communication was associated with increased study drop-out rates [58]. This recurring contact could be in person (e.g., accompanying a mother to a Women Infants Children appointment) [59], via mail (e.g., holiday or birthday cards for child participants) [47,59], or through communication with the caregiver (e.g., phone, email, or social media connection) [38,47]. Finally, ensuring accurate and up-to-date contact information of the caregiver as well as identifying alternate relations that can be reached and making repeated attempts to connect were noted to be essential for successful retention in longitudinal research [38,51,59,60].

#### Timing of consent

The timing of and approach to consent can be important in optimizing the effectiveness of recruiting caregivers and their children into a study, particularly in a study like HBCD which plans to recruit pregnant and postpartum individuals and those with young children. Attempting study enrollment during the early stages of parenting was found to be overwhelming to some new parents and hindered recruitment rates [57]. However, minor shifts in approaches, such as obtaining verbal consent in the immediate postpartum period for noninvasive collection of biospecimens in newborns (with the full informed consent and biospecimen linkage to clinical data within the following week), can minimize caregiver burden and be an effective strategy for recruiting newborns and their families [61]. Similarly, poor timing of consent efforts was also found to be a reason for parental disinterest in research participation in scenarios outside the newborn period, specifically when a child was admitted to the hospital and the decision to enroll in a study was time-sensitive [41]. Allowing caregivers adequate time to make their decision to participate, including time for a parent to discuss the proposal with their partner or other family members, increases the likelihood of study enrollment [38,62].

#### Understanding the study

Another aspect that should be considered when approaching enrollment and retention in longitudinal studies is ensuring the caregivers understand the study. Knowledge of a study facilitates recruitment and retention of caregivers and their children through the follow-up period [58,62]. Specifically, comprehending the purpose and importance of the research [38,58], knowing the study procedures and time commitment [38], and having access to the study results [38,53,63] were noted to be important to individuals approached for recruitment. Additionally, knowing how their child was contributing to a larger goal or “greater good” was a motivator for a caregiver’s ongoing study participation [53], and caregivers were more likely to continue research participation if they recognized the study could potentially help their child or other children [62]. Retention was also enhanced when the caregiver felt part of the working alliance and understood their privacy was important to the study team [64], while apprehension over privacy, confidentiality, and transparency negatively influenced their decision to take part in a study [38,63].

Conversely, not fully comprehending the study procedures or the potential benefits of the research was shown to negatively impact recruitment [41]. Caregivers were also more likely to decline participation if they perceived there was a possible risk to their child by taking part in the study [38,62], if they were concerned over the invasiveness of biospecimen collection, or if the study would compromise their child’s autonomy [38,63].

#### Minimizing burden

Several studies demonstrated that reducing the burden on families was important in fostering participation in research. Families’ busy schedules, work conflicts, travel distance and transportation issues, lack of childcare, and frequent changes in contact information all served as barriers to recruitment and retention of caregivers and their children into research investigations [38,42,45,54,59]. For low-income families in particular, securing transportation, having childcare, and time constraints were found to impact retention [59]. To address these logistical barriers, conveniences such as after-hours and weekend calling [44,51], study-provided transportation [38] and/or covering transportation/parking costs associated with study appointments [51], and providing childcare during data collection improve participation and long-term retention [38].

Families can also feel burdened by the data collection process. Study drop out increased when the study was considered too time consuming, questions were perceived as redundant, or the response for data collection was felt to be burdensome [60,65]. To address this issue, study teams can utilize programs that were already in place to reduce participant burden caused by data collection [61]. Specific examples include leveraging existing databases and utilizing electronic medical records to extract necessary information (instead of eliciting it from the participants)[50,54,61,66], completing comprehensive surveys and assessments at the initial visit to decrease the number of follow-up visits for data collection[61], and offering study assessments via phone or other remote options (rather than in-person only) [67].

Participant retention can also be enhanced by using the conveniences of modern technology for communication and data collection. Short message service (SMS) text messaging and email communication were shown to be a preferred modality of communication for many participants and can be used for greetings, reminders, contact information updates, and even some data collection over the course of a study while being mindful of securing confidentiality and privacy of protected health information [68,69]. Digital reminders and multiple SMS text messages were also found to increase clinic attendance [69]. Finally, study websites were proposed as a strategy to keep caregivers up to date and engaged in the research [57], a strategy proven effective in the general retention literature [70].

#### Incentives

Incentives and compensation for time and effort are important for participating caregivers [38,51,60], as is the timeliness of the reimbursement [51]. Most commonly, financial compensation (ranging from $20-$75 per session in the reviewed studies) was found to be useful in promoting participation and diminishing the burden of time, lost work, and/or travel required for involvement in the investigation [38,60,69]. Financial compensation was also noted to be useful in encouraging health care providers to refer qualifying patients to the research team [44,52]. Access to free healthcare services was observed to be a research motivator for families from lower socioeconomic backgrounds [57]. For some individuals, contributing to knowledge in the area of study or helping others acted as an important, nonmonetary recruitment incentive [62].

#### Study population

One of the more difficult aspects of recruitment and retention surround factors that are largely unmodifiable. Maternal lifestyle behaviors, including smoking, alcohol use, and working outside the home during pregnancy, were predictive of study drop-out [56]. Similarly, parental demographic variables, such as younger age, unmarried, lower household income, less formal education, un-/under-employed status, and self-identification as a minority, were strong predictors of caregiver–child attrition in longitudinal studies [56,71]. Furthermore, certain populations were found to be less likely to participate in research, such as the socioeconomically disadvantaged, underrepresented minorities, and those individuals for whom a cultural barrier was present [54,72].

Investigators seeking to recruit an inclusive and representative panel of participants will need to be aware of special challenges that may impact an individual who is a minority or holds particular cultural beliefs. Specifically, for some minorities, especially those with limited English proficiency (LEP), lack of study interpreters and availability of printed materials in multiple languages was noted to be an obstacle to successful recruitment [40,73]. For populations with differences in cultural customs and beliefs, mistrust of medical experts was found to interfere with participant enrollment [73]. However, these challenges can be overcome with proper planning. Several studies found that involving community leaders and establishing research partnerships with community organizations serving the population of interest increased minority caregivers’ willingness to participate in research studies [40,59,73]. For minority caregivers with LEP, having a bilingual researcher or staff who spoke the language of the family or interpreter services was essential in engaging participants and promoting retention [51,73]. Hiring culturally competent and culturally sensitive staff with strong interpersonal skills to work with families was helpful with retaining caregivers and their children from the minority backgrounds [51].

Investigators need to also consider nontraditional families in which a father or grandparent may have the primary custody of a child. While most studies focused on recruitment of mothers and their children, some specifically addressed engaging fathers or grandparents in research with their children or grandchildren, respectively. For fathers, online crowdsourcing appeared to be a valuable tool in encouraging paternal participation in research investigations [74]. While fathers were more apt to drop out early in prospective studies, they were more likely to remain for the duration of the study if they attended the second visit, suggesting that study team efforts should focus on early engagement with and retention of fathers [74]. Grandparents were more likely to participate in research if informed about the study by an already-involved parent [75]. Grandparent recruitment also benefited from person-to-person communication (vs email) and the use of different questionnaire modalities (pencil/paper options vs only computer/tablet) [76].

## Discussion

This scoping review synthesizes the data on facilitators and barriers to recruitment and retention of young child–caregiver dyads, providing information that could help improve research engagement of this population in longitudinal cohort studies. The impetus for this review was to prepare for a multicenter prospective, longitudinal study evaluating the complex interplay between innate and extrinsic factors in child development, particularly in children with previous *in utero* substance exposure, as set forth by the NIH HBCD study [20]. The HBCD-funded cohort of children will be intensely studied and followed from *in utero* through early childhood. To accomplish this goal, the HBCD study teams will need to successfully recruit and retain a large cohort of pregnant persons, caregivers, families, and their children using the informed approaches set forth in this review.

The methodologic approach was a key factor in the success or failure of recruitment and retention of caregivers and children in the studies included in this review. The HBCD study aims to oversample for children with prenatal substance exposure, and, therefore, the study team will need to deliberately consider how the selected recruitment and retention techniques may apply differently to persons affected by substance use. Results from previous, longitudinal studies on prenatal exposures to marijuana [77,78], cocaine [79], and opioids[79–81] can provides useful guidance on navigating these approaches.

Pregnant and parenting individuals with SUD are a highly stigmatized and vulnerable group[22–24] who have high rates of coexisting mental health disorders [82], higher incidences of mental health diagnoses in the first postpartum year [83], histories of trauma [84], and limited social supports [22]. Allowing researchers access to their personal lives involves real risks to these individuals with 33 states now having some level of punitive policies in place for substance use during pregnancy despite contrary guidance from professional societies and federal agencies [26]. It is critical that researchers have a good understanding of each state’s laws and local policies on mandated reporting and differences of such reporting within the research versus clinical settings. Furthermore, longitudinal research can present ethical challenges due to its continuous data collection and its study of vulnerable populations. This concern is particularly relevant in a study that aims to follow persons with both SUD and their children. For individuals affected by SUD, issues may arise related to sustained relationships with research staff, potential for relapse and change in clinical status, and interactions with the legal system [85]. Similarly, children participants are considered vulnerable given their inability/limited ability to provide informed consent, their observation over long periods of time, and the potential stress and discomfort of research tasks [86].

Special precautions need to be taken to ensure the safety of these persons and their children. The HBCD’s broader advisory committees, in collaboration with local community partner-advisors, offer specific recommendations on how to address concerns around the law, the community, relationships, and personal needs and challenges that pertain to individuals with SUD [87]. Additionally, ethically informed research protocols, staff training in monitoring and supervision of participants, and debriefing after critical events aid in protecting individuals with SUD [85]. Finally, to safeguard children, researchers should be mindful of infant and child experiences in research studies, and both investigators and participants should conceptualize consent as a continued process in longitudinal research [86].

There were several specific methodologic strategies identified in this scoping review that can aid investigators in engaging caretakers and their children in research studies. The setting of the recruitment played a key role in the success of study enrollment of caregiver–child dyads. While labor-intensive, face-to-face recruitment largely performed better than more impersonal strategies, such as mass emailing [39,88]. However, this assertion was not always the case[43] and emphasizes the role of various additional circumstances in a caregiver’s decision to participate in a research study. In every approach, building a relationship with the participant was crucial to successful recruitment and retention. This connection was particularly important for persons with a history of SUD [64,87].

Though less successful in recruitment, web-based tools have become increasingly popular in research secondary to their ease of use [34,35,38,39,45,46]. Online strategies differ greatly in their uptake by various populations, with social media platforms like Facebook™ achieving recognition for better ability to recruit underrepresented populations [35], and internet-based approaches performing better at engaging older and more educated individuals [89]. Additionally, while online modalities like Facebook™ have effort advantages in early recruitment, participants enrolled through this strategy have poorer retention rates compared to the more time-intensive provider recruitment [35]. Paid media, including commercials and online advertisement, also performed well, though at an increased monetary expense [38,44].

While convenient and now widely used, these digital methods require special privacy and confidentiality considerations, particularly for individuals with SUD given the various punitive laws around pregnancy and substance use in several states [26]. Data from websites and apps can access and store large amounts of data, and protocols that use these modalities need to describe how the website or app functions so that Institutional Review Board (IRB) members and participants may understand the relationship the website/app will have with the research [90]. Researchers should contact their IRB during the planning stage as many institutions now have guidelines in place to ensure computer- and internet-based research protocols address fundamental risks (violation of privacy, legal risks, and psychosocial stress) and provide the same level of protection as other research involving human participants [91].

The personnel in the position of approaching participants for enrollment also played a significant role in the success of recruitment and retention of caregivers and their children. Medical provider recruitment outperformed several other types of personnel in engaging a participant, but was noted to be burdensome for the clinician due to competing workloads and potential encroachment on the provider–patient relationship [44,50]. However, it appears that this barrier could be partially overcome by ensuring the providers were not responsible for providing detailed study information and by offering reimbursement to providers for their time in recruiting patients [44,52]. In contrast to physician *recruitment*, physician *referral* after the patient reviewed study-related reading material was found to be effective with less burden on the provider, but it required four times as many attempts at contact by the research team than a referral from a family friend, potentially limiting the utility of this approach by the research team [44].

Though the initial introduction of a research investigation by a member of the healthcare team generally produced higher enrollment [38,41,54], using clinical nurses to explain the study and refer interested patients to the research team failed to effectively recruit participants, and causing the research staff to contact families directly [54]. In this study, nurses felt unprepared to obtain consent directly due to inability to provide sufficient study information and clinical time constraints, an issue that was echoed in other investigations as well [44,50]. Response rates may have been improved by providing a family with a decision aid [37], designating a nurse whose sole role was communication with the participants [53], or, if feasible, assigning staff exclusively dedicated to recruiting patients [51].

Timing of approach also plays an important part in the success of caregiver–child recruitment into research studies. Efforts at enrolling during the initial phases of child-raising was often noted to be difficult as caregivers were preoccupied by more immediate issues of caring for a newborn [57]. When interviewed, caregivers indicated that other times, such as during pregnancy or after the first several weeks of the baby’s life, might be more effective in recruiting families [57]. Additionally, when a child is admitted to the hospital, as would be the case for infants being observed or treated for opioid withdrawal, parental stress was found to negatively influence a caregiver’s willingness to participate in a research investigation [41]. In both these scenarios, a potential solution may be to consider initial verbal consent followed by formal, informed consent at a later, less-stressful time [61].

The burden placed on the caregiver and child during the actual execution of the study can be overlooked and addressing these issues proactively can increase the successful recruitment and retention of caregivers and their children into longitudinal research studies. Families’ busy schedules, work conflicts, transportation issues, and lack of childcare were frequent barriers to caregiver-child recruitment and retention [38,42,45,54,59]. These challenges are particularly pertinent for persons with SUD as they often experience socioeconomic difficulties such as unemployment, housing insecurity, and transportation barriers [22,25]. Similarly, for low-income families these issues were especially challenging as were lack of reliable phone service and the inability to provide consistent contact information due to unstable life circumstances [59].

To minimize these inconveniences for caregivers and their children and encourage enrollment and retention, several approaches have been noted to be helpful. Specific solutions include incorporating evening/after-hours and weekend calling [44,51], offering participation through phone calls when feasible [67], allowing flexibility in scheduling or who brings the child participant [38,60], providing childcare during data collection [38], and covering transportation and parking costs[51] or providing transportation [38]. For families struggling with reliable communication, successful strategies to promote study retention include obtaining alternative contacts and updating them regularly[51] and maintaining regular contact in the follow-up period to avoid possible attrition [38,53,57,59,92]. Finally, incentives and compensation for time and effort can offset the burden of the study protocol [38,51,60]. However, special attention must be paid to the issue of coercion, especially in disadvantaged and vulnerable populations such as those with SUD. Disparities in income, paid time off, childcare, and transportation challenges make it difficult to determine a universal incentive for participant time and effort [93]. Helpful solutions to this challenge include seeking input from community advisory boards and considering a sliding scale for compensation so that lower income populations receive higher compensation in an effort to overcome barriers to participation that may not be present for those with higher incomes, more stable homes, and access to more resources [94].

Several studies pertaining to recruitment and retention of caregiver–child dyads specifically focused on minority families, and inclusion of underrepresented minority populations in research is a priority for the NIH as enrollment barriers are particularly prevalent in minority communities [95,96]. For some minority families, cultural and linguistic barriers were found to negatively affect enrollment and study retention [40,57]. These challenges can be overcome but require dedicated efforts from the research team in securing bilingual staff and interpreter services[73] and garnering support from community gatekeepers in order to engage the residents of their community [40,59,73]. Further, peer navigators have also been shown to increase engagement of vulnerable, hard-to-reach populations [97,98].

There are several limitations to this literature review. First, the approach for the review was not a systematic review or meta-analysis, and as such, can be prone to author bias and interpretation. However, the review was undertaken in a standardized fashion, with clear inclusion and exclusion criteria, and any uncertainty over an article’s eligibility for inclusion was reconciled by discussion among the study team. In an effort to identify factors that would be helpful in the development of a longitudinal cohort starting in early childhood or infancy, the inclusion criteria mandated that the article reviewed involved children 6 years of age or younger. Therefore, the facilitators and barriers to recruitment and retention identified in this review may not be broadly applicable to engaging caregivers and their older children in research. Additionally, the scoping review utilized PubMed database; articles indexed outside PubMed may have been missed. Despite these limitations, this study entailed a structured and purposeful review and synthesizes the literature on recruitment and retention of caregivers and their children into longitudinal research. Consistent with a recent systematic review on recruitment and retention strategies of pregnant persons [99], this study emphasizes that utilization of a variety of recruitment and retention methods is more likely to achieve a large, population-representative cohort [44,72], The information from this review will be useful in future, prospective cohort studies aiming to study the specific population of caregivers and their children.

## Conclusion

Numerous factors affect engagement of caregivers and their children in observational cohort studies. Our findings indicate there is no clear, single strategy that is universally effective in engaging caregivers and their children, and multiple approaches should be considered and tailored to the specific populations of interest in the HBCD study. The information here may inform future study designs aimed at the prospective analysis of long-term outcomes in caregivers and their children.

## Supporting information

Corr et al. supplementary material Corr et al. supplementary material

## Supplementary material

The supplementary material for this article can be found at https://doi.org/10.1017/cts.2024.624.
