# Supplementary material for: Recruitment and retention strategies to promote research engagement among caregivers and their children: A scoping review
Source: J Clin Transl Sci. 2024 Nov 8;8(1):e194. doi: 10.1017/cts.2024.624 (PMC11626585; doi:10.1017/cts.2024.624)
Supplement: Corr et al. supplementary material [file S2059866124006241sup001.docx]

**Supplementary Table 1. Electronic Database Search Strategy**

| **Step** | **Search Term** |
| --- | --- |
| 1 | Recruitment [tiab]^*^ AND parent [tiab] OR parental [tiab] OR maternal [tiab] OR paternal [tiab] OR mother [tiab] OR family [tiab] OR pregnant [tiab] OR postnatal [tiab] OR antenatal [tiab] OR prenatal [tiab] OR neonatal [tiab] OR neonate [tiab] OR newborn [tiab] OR infant [tiab] OR baby [tiab] OR infancy [tiab] OR toddler [tiab] OR child [tiab] OR children [tiab] OR pediatric [tiab] |
| 2 | Retention [tiab] AND parent [tiab] OR parental [tiab] OR maternal [tiab] OR paternal [tiab] OR mother [tiab] OR family [tiab] OR pregnant [tiab] OR postnatal [tiab] OR antenatal [tiab] OR prenatal [tiab] OR neonatal [tiab] OR neonate [tiab] OR newborn [tiab] OR infant [tiab] OR baby [tiab] OR infancy [tiab] OR toddler [tiab] OR child [tiab] OR children [tiab] OR pediatric [tiab] |

^*^tiab = search title and abstract
